# Multiple uncertainties require a change of conservation practices for saproxylic beetles in managed temperate forests

**DOI:** 10.1038/s41598-018-33389-9

**Published:** 2018-10-08

**Authors:** Andrey L. D. Augustynczik, Rasoul Yousefpour, Marc Hanewinkel

**Affiliations:** Chair of Forestry Economics and Forest Planning, University of Freiburg, Tennenbacher Str. 4 (2. OG), D-79106 Freiburg, Germany

## Abstract

In Europe, intensive forest management has severely compromised the habitat of forest insects, especially saproxylic beetles, due to the removal of deadwood and veteran trees. The loss of insect diversity may disrupt ecosystem functioning and affect the provision of important ecosystem goods and services in the future. Here we propose a novel approach for the implementation of conservation policies, by optimally allocating forest reserves and deadwood islands under multiple sources of uncertainty and minimizing economic risk. We use the saproxylic beetle *Lucanus cervus* as umbrella species, requiring that deadwood islands were spaced within its dispersal capacity. We show that current management and conservation practices are increasingly inefficient under changing environmental conditions and that the consideration of uncertainty requires a major expansion of conservation areas. Moreover, our results indicate that a strong diversification of management regimes, with a focus on selection forest systems, is required to reduce economic risk of forest management. We conclude that the integration of uncertainty into conservation planning may reduce the trade-off between production and conservation objectives in forest landscapes and is key to increase the efficiency of forest management in the future.

## Introduction

Forest biodiversity has been declining worldwide at alarming rates and the implementation of conservation policies targeting at mitigating this trend is crucial^1^. Biodiversity plays a central role in ecosystem functioning, affecting the efficiency of energy use and biomass production in forest ecosystems^2^. Consequently, biodiversity loss poses a major threat to human well-being, as it affects the provisioning of ecosystem goods and services, such as wood production, climate regulation, erosion control and the regulation of water quantity and quality^3^. Moreover, in the face of climate change impacts and the expected increase in forest disturbance activity^4^, maintaining biodiversity becomes decisive for the recovery of ecosystem functioning after natural hazards. Evidence shows that biodiversity may increase the resistance of ecosystem to climate extremes^5^, supporting key processes, such as pollination and nutrient cycling^6^. Hence, biodiversity decline may trigger substantial economic and ecological losses in the future and the correct valuation and implementation of efficient conservation policies is urgent.

In Europe, 85% of forests are managed and used for wood production^7^. This high degree of utilization affects the habitat of several taxa and requires optimized conservation solutions that promote the multifunctionality inherent to these ecosystems. Currently, insect decline is one of the most pressing issues to be addressed by conservation policies, especially due to the potential severe economic impacts through the loss of pollinators^8,9^.For example, Hallmann *et al*.^10^ reported a shocking 76% seasonal decline in flying insect biomass in Germany over the last 27 years. Saproxylic beetles represent a large share of forest biodiversity in temperate Europe and recent estimates indicate the existence of over three thousand saproxylic beetle species^11^. These insects depend on dead or decaying wood in at least one phase of their life cycle and directly contribute to nutrient cycling and pollination^12^. Despite the crucial role for ecosystem functioning, intensive forest management has severely compromised their habitat through the removal of deadwood and undersupply of veteran trees^13^. According to the IUCN Red List, in Europe and nearly 18% of saproxylic beetles are presently classified as threatened. The most recent assessment points out that 0.7% of the species are critically endangered, 7.4% endangered 5.4% vulnerable and further 13% of the species are near threatened^11^.

A variety of conservation strategies have been proposed in order to ameliorate forest biodiversity loss, e.g. the increase in the number of protected areas and the improvement of the connectivity between forest reserves^14^. During the past decades, retention forestry has been a relevant policy for supporting biodiversity in managed forest landscapes. Retention forestry practices were proposed initially for temperate and boreal forest ecosystems in northwestern North America as a cost-effective alternative for balancing wood production and biodiversity conservation^15,16^. Retention forestry consists of the maintenance of structural elements relevant for biodiversity that are scarcely available due to forest management, such as old trees and deadwood, in order to facilitate the persistence of forest biota^16^. The implementation of retention forestry policies has shown positive impacts on community composition, species richness and abundance worldwide^17^. Among the most relevant retention forestry actions that may benefit saproxylic organisms is the creation of the so-called “deadwood islands”. Deadwood islands, are areas typically around 1 ha (ranging from 0.5 to 20 ha) set aside from management that may act as stepping stones for species dependent on large deadwood amounts and old trees in the managed forest landscape^18,19^. Deadwood islands can increase the habitat suitability and sustain higher levels of species richness and abundance^20,21^ and numerous studies show positive impacts of such retention patches on forest biodiversity^18,20,22^.

The establishment of forest reserves and the allocation of deadwood islands in managed forests, however, must take into account economic aspects of forest management and the multiple use of forest landscapes. Managers need to select cost-effective conservation alternatives to reduce the trade-off between wood production and forest biodiversity conservation, harmonizing forest and conservation planning at strategic and operational levels. Thereby, we can find an optimal layout of forest reserves and deadwood islands in the landscape to support saproxylic beetles and other deadwood-dependent organisms with the least trade-off to forest profitability.

A major challenge for the planning and implementation of forest management and conservation actions are the multiple sources of uncertainty posed to forest ecosystems by changing environmental and economic conditions. Due to the long life span of the trees, these sources of uncertainty may significantly affect decision-making (e.g. Passalodos-Tato *et al*.^23^) and change the relationships between conservation and production objectives in forest landscapes. Arguably, the most significant sources of uncertainty to forest and conservation planning are the uncertainty in climate development, occurrence of forest disturbances and economic conditions. Fluctuations in precipitation regimes, temperature, and atmospheric CO_2_ concentration are projected to modify forest growth patterns and species distribution ranges, demanding a timely and adequate response from forest management (e.g. Bonan^24^; Hanewinkel *et al*.^25^; Reyer *et al*.^26^). The occurrence of disturbances and its expected increased activity due to climate change produces major impacts on the functioning and structure of forest ecosystems^4,27^. Thus, it influences not only the commercial value of forests, e.g. by requiring an anticipation of stand harvesting and reduction of wood value, but the provisioning of ecosystem services as well, e.g. by releasing large amounts of carbon and exposing soils to erosion and nutrient loss^28^ Finally, uncertain development economic conditions, namely the development of wood prices and interest rates, may affect the behavior of decision-makers and harvesting patterns in forest landscapes^29^.

To account for the interactions between ecological and economic responses of forest ecosystems under uncertainty, coupling economic and ecological models is key to provide sound conservation and forest management alternatives. It is still unclear, however, how to operationalize conservation actions efficiently and how to integrate these multiple sources of uncertainty into the decision-making process. Here we propose a novel approach to tackle these issues through an efficient allocation of conservation practices, targeted to support saproxylic beetles under multiple sources of uncertainty in a typical managed forest landscape in central Europe. We consider the optimal creation of new forest reserves and the allocation of deadwood islands in the forest landscape with minimum cost, taking into consideration the dispersal capacity of the indicator species *Lucanus cervus*^30,31^, which is also typical for other indicator species such as *Osmoderma eremita*^32,33^.

To reinforce the optimal solution against climate, economic and disturbance uncertainty, we provide a robust optimization model using a safe tractable approximation scheme, based on a framework proposed for complex problems in operations research^34^. In our robust optimization, we maximize the Value-at-Risk (lower 5% quantile of the forest profitability distribution) under uncertain ecological and economic conditions to dilute the economic risk of forest management. Moreover, we investigated the role of uncertainty in the landscape design and conservation actions through the robust optimization model and identified the caveats of neglecting the multiple sources of uncertainty inherent to forest ecosystems and decision-making.

## Results

### Optimal allocation of conservation areas under uncertainty

We created an optimization model for an optimal allocation of deadwood islands and forest reserves within an intensively managed forest area, harmonizing forest and conservation planning at strategic and operational level, under deterministic and uncertain conditions. Our model successfully created new forest reserves and connected them through a network of deadwood islands, thus enhancing the probability of species persistence in the landscape.

**Figure 1:**
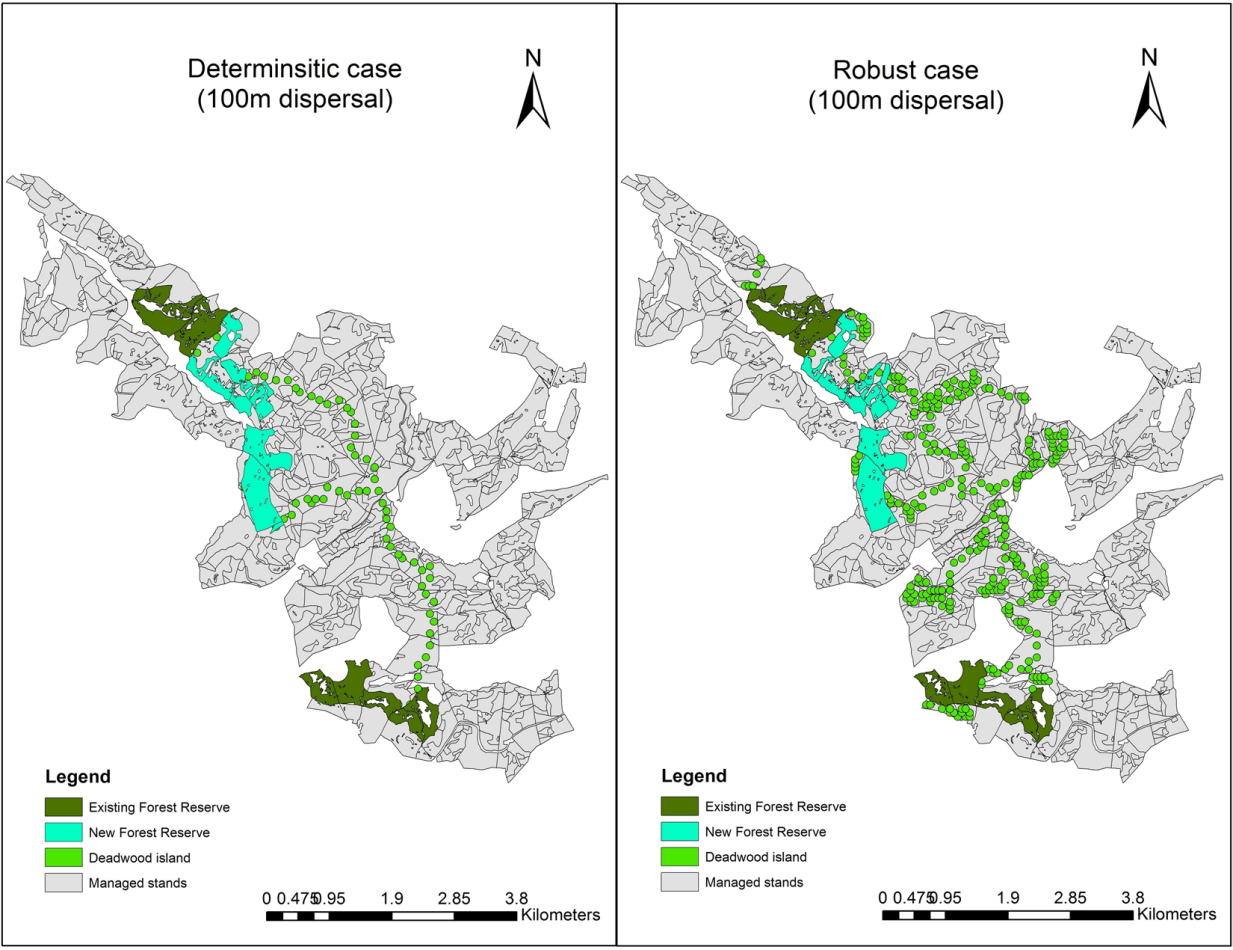
Optimal allocation of forest reserves and deadwood islands in the forest landscape (2534 ha with 145 ha of existing forest reserves). The left panel shows the result for the deterministic case (RCP4.5), considering a 100 m dispersal capacity and the right panel shows the same outcomes for the robust case, taking into account multiple sources of uncertainty. The map was created using ArcMap 10.3.1 (http://desktop.arcgis.com/en/arcmap/).

We perceived that by mitigating the economic risk of forest management in the robust optimization model, the trade-off between forest profitability and biodiversity conservation was reduced and there was a major expansion in the deadwood islands network (Fig. 1). While the optimal habitat network configuration showed a rather regular pattern for the deterministic case, disregarding the multiple uncertainty sources (left panel in Fig. 1), the consideration of uncertainty favored the expansion of the reserve network (right panel in Fig. 1) and several stands had their whole area designated as a deadwood island. This indicated either a negative Net Present Value (NPV) yielded by such stands, regardless of the management regime applied, or high risk levels due to disturbances and economic uncertainty. In this regard, the expected profitability of the stands was inferior to the risk they added to forest profitability, decreasing the Value-at-Risk (VaR) of the management portfolio. Therefore, it was beneficial to include them in the habitat network.

The configuration of forest reserves remained similar for all dispersal distances considered, taking into account both deterministic and robust cases, setting aside old-growth beech stands (see details in Supplementary 2) and that the most cost-effective option for enhancing habitat connectivity did not coincide with the shortest path between forest reserves. Therefore, the consideration of local forest characteristics was key for reducing the costs of the habitat network and to reduce the trade-off between competing conservation and production objectives in the landscape matrix.

### Optimal management portfolio and wood production

**Figure 2:**
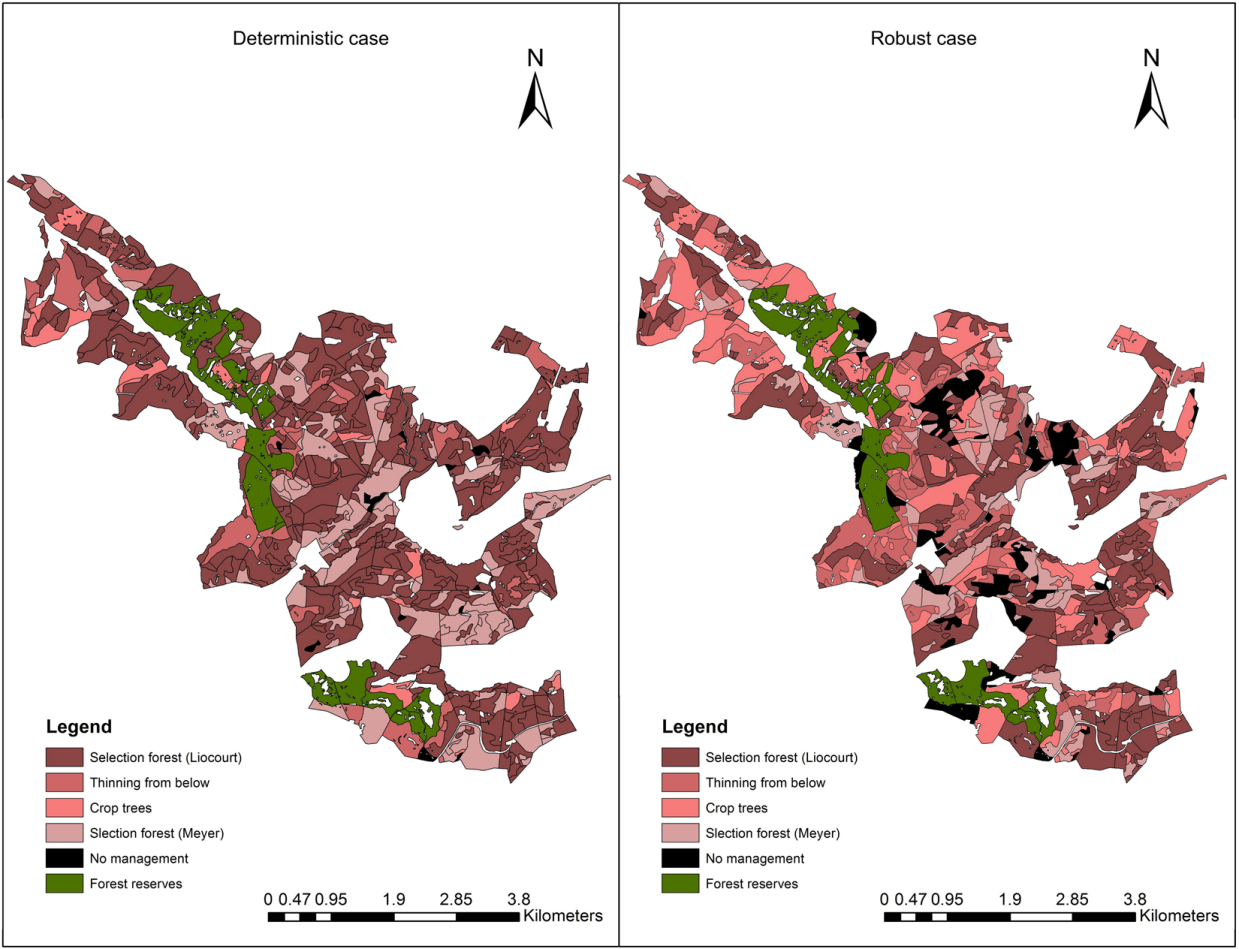
Management portfolio for the deterministic (left hand side) and robust (right hand side) solutions, taking into account a 100 m dispersal capacity scenario and RCP4.5 for the deterministic case (Fig. 1). The red color gradient indicates the intensity of forest management in terms of early harvestings, where “Liocourt” and “Meyer” indicate selection forest systems, and the black color indicates no management (for details on the management regimes see Supplementary 3). The map was created using ArcMap 10.3.1 (http://desktop.arcgis.com/en/arcmap/).

The inclusion of multiple sources of uncertainty in our planning problem led not only to substantial modifications in the configuration of the habitat network, but on the optimal management portfolio as well (Fig. 2). As a result, it was necessary to adapt forest management to novel conditions in order to safeguard production objectives of forest management, namely forest profitability and wood production. In the deterministic case, considering the intermediate climate trajectory RCP 4.5 (left panel of Fig. 2), we perceived a dominant shift from crop-trees to selection forest management systems, with a balance between intensive harvesting in the dark red-colored stands and the lowest wood utilization in the light red-colored units. Thereby, it was possible to substitute slow growing forest areas, by faster growing stands, increasing forest profitability, while allowing fast growing stands to capitalize in areas with reduced utilization. With the inclusion of uncertainty, (right panel of Fig. 2) it was beneficial to diversify the management portfolio and increase the share of early harvestings, reducing the amount of capital at risk in case disturbances occurred and decreasing the uncertainty introduced by price development. Moreover, in order to counterbalance the increased harvesting rate, and prevent the reduction in standing stock required in our model, the proportion of stands with no harvesting interventions substantially increased (black areas in Fig. 2).

**Figure 3:**
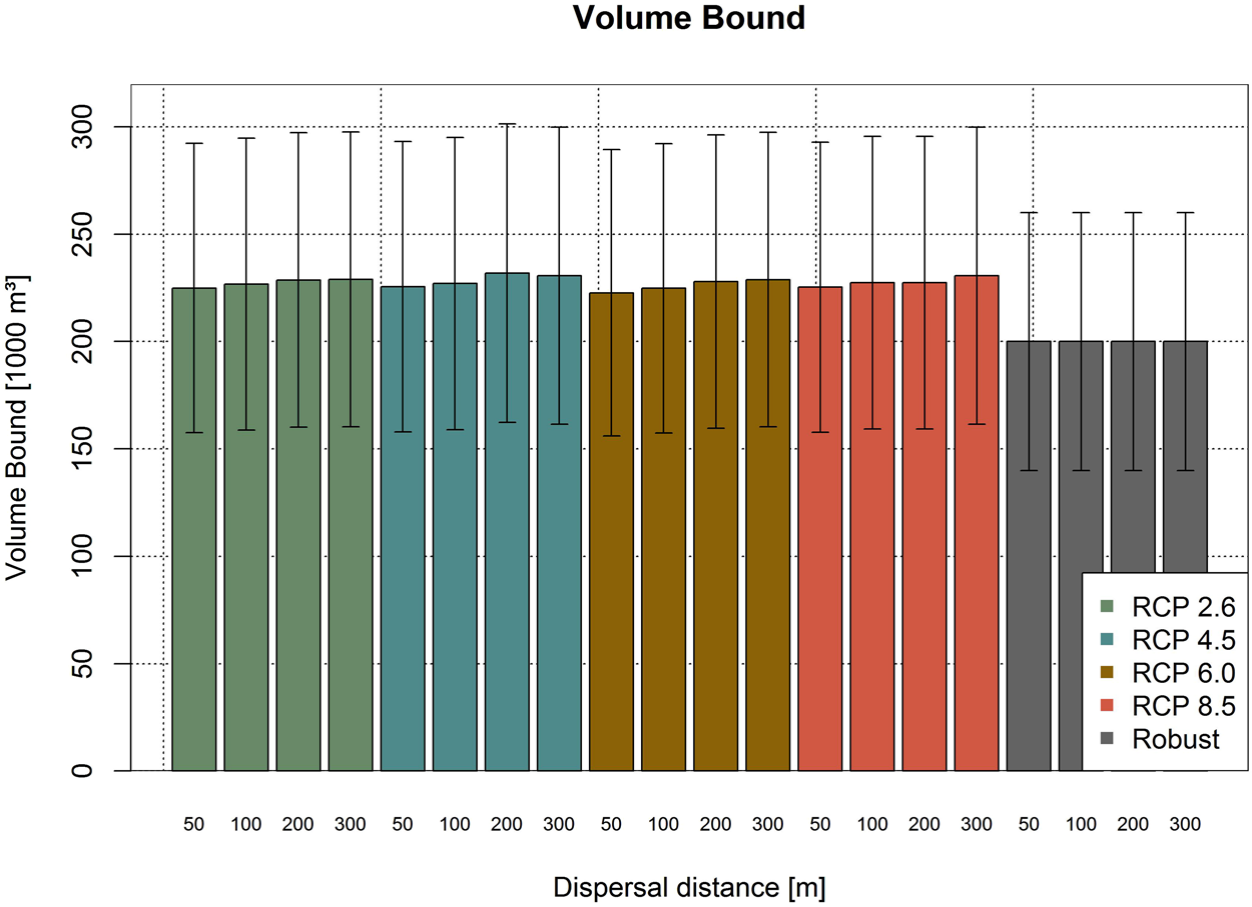
The figure shows the optimal volume bound, i.e. the wood volume harvested each 10-years period may not vary more than 30% of this threshold. The upper and lower harvesting limits each 10-year period are indicated by the error bars. The figure was created using RStudio 1.1.456 (https://www.rstudio.com/).

The shift in the forest management strategies affected the harvesting patterns in the forest area. Therefore, to avoid high fluctuations in wood production along the planning horizon, we established wood production bounds in our model, i.e. limits on the amount of harvested wood in each 10-year simulation period. Thereby, we aimed to sustain wood production at desirable levels and avoid commercialization problems arising from over- or underproduction. The volume bound obtained for each climate trajectory and dispersal capacity is shown in Fig. 3. We defined that the harvested wood volume in each 10-years period could not vary more than 30% compared to the volume bound, which varied from 200 thousand m³ to 240 thousand m³ in our solutions. This represents a utilization rate ranging from 7.5 to 9.7 m³/ha/year over the whole area. Moreover, for the robust case we enforced that the bound should be maintained above 200 thousand m³. The inclusion of the habitat network slightly reduced the volume bound due to the creation of deadwood islands, as less productive area was available. The climate trajectory did not affect substantially the volume bound for the deterministic cases, with the threshold neighboring 220 thousand m³. Conversely, when uncertainty was added in the robust solution, the bound reduced to 200 thousand m³. This behavior arose from the reduction in the productive area, due to the increase in the number of deadwood islands (Fig. 1) and the requirement to accommodate the lower harvesting rates at the later periods of the simulation period, as the proportion of early harvestings increased for the robust case.

### Economic impacts of habitat selection and the role of uncertainty

**Figure 4:**
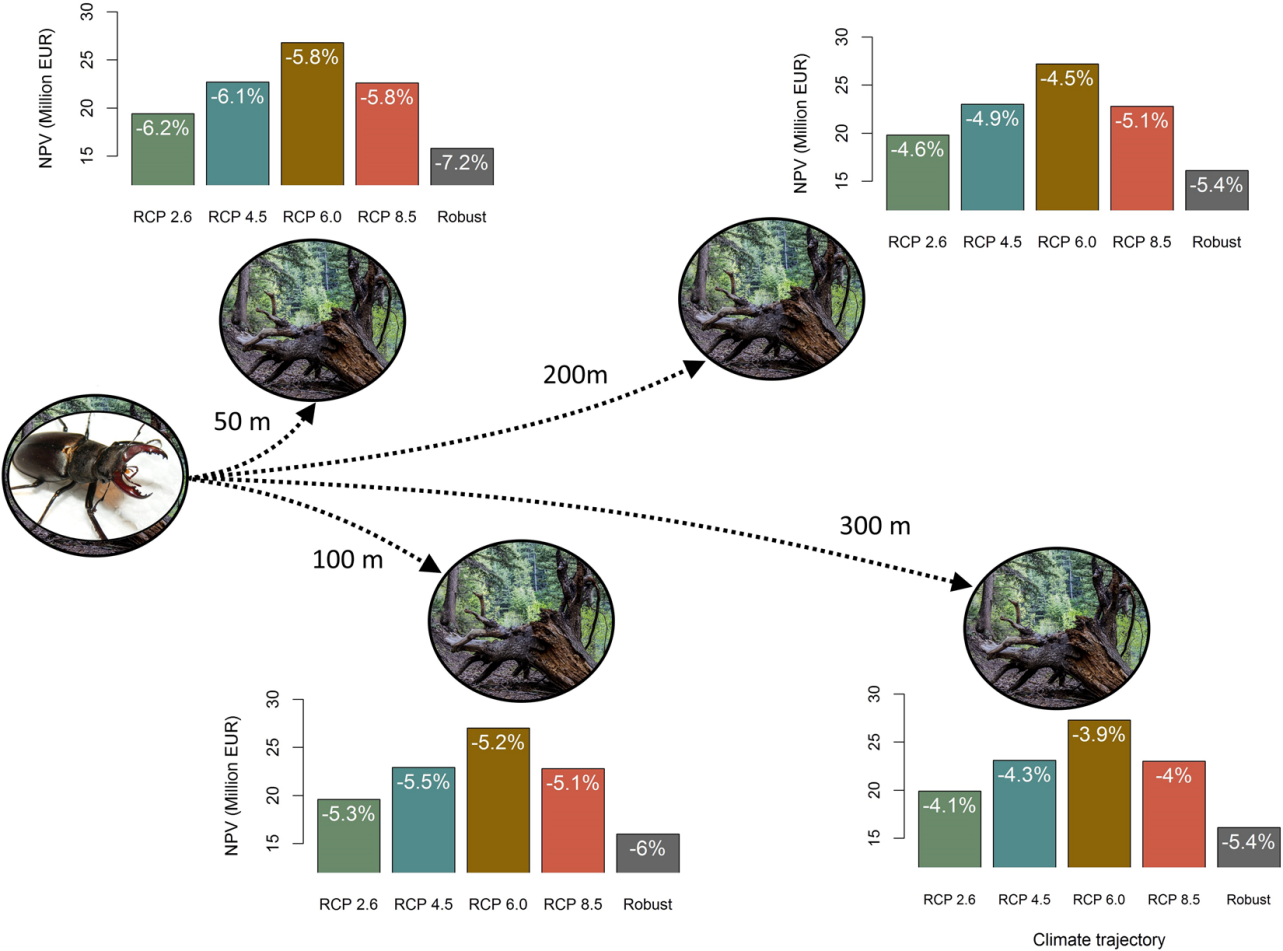
The figure shows the forest profitability for all four dispersal capacities considered (50, 100, 200 and 300 m), for each climate trajectory considering the deterministic case and the expected net present value (NPV) for the robust case, taking into account all climate trajectories, economic and disturbance uncertainty. Moreover, the relative NPV reduction compared to the baseline scenario, without the creation of the habitat network, is displayed inside the corresponding bar. For the robust case, the reduction corresponds to the loss in the Value-at-Risk. (Sources: https://pixabay.com/pt/vida-selvagem-animais-insetos-87168/; https://pixabay.com/pt/%C3%A1rvore-ra%C3%ADzes-ver%C3%A3o-1391055/). The figure was created using paint.net version 4.0.12 (https://www.getpaint.net/).

The economic output of forest management showed sensitivity both to climate conditions and the dispersal capacity considered. Figure 4 displays the economic outcomes, in terms of the Net Present Value (NPV) for each climate change trajectory and for the robust case under different dispersal distances. The respective loss in NPV due to the creation of the habitat network for saproxylic beetles is shown in white, inside the bars. Assuming perfect knowledge of future climate and economic parameters, the total NPV ranged from 21 to 28 Million EUR depending on the climate trajectory. The profitability under low climate change intensity (RCP 2.6) displayed the poorest outcomes across all climate trajectories, whereas the revenues increased under moderate climate change (RCP 6.0), thus indicating that CO_2_ fertilization was not the only driver of the increased profitability in higher climate change intensity, but rather a combination of climatic factors, including temperature, vegetation length and precipitation.

Taking into account the selection of the habitat network for biodiversity, the creation of forest reserves and deadwood islands led to a decrease in NPV ranging from 3.9 to 6.2% in the deterministic case. The lowest economic impacts in relative terms occurred under RCP 6.0, whereas the largest effects were observed for RCP 4.5 (on average of all dispersal capacities). The creation of forest reserves contributed the most to the total costs of the habitat network in the deterministic case, especially for large dispersal capacities, since only a few islands (~20) were created and the NPV loss amounted to 4%. For low dispersal capacity (e.g. 50 m) there was also an important contribution of the deadwood islands increasing the NPV loss from approximately 4% to 6% (Fig. 4). Therefore, increasing the dispersal capacity reduced the total impacts on the objective function, as noted for all climate change trajectories. The increase in dispersal capacity from 50 to 300 m caused a concurrent increase of 2% in the objective function on average of all climate trajectories.

The consideration of uncertainty showed surprisingly important impacts on our optimization model (Fig. 4). We perceived a strong reduction in the expected NPV due to the perturbations in wood prices, interest rates and forest disturbances, leading to substantially lower profitability levels than the ones achieved under deterministic conditions. The robust solution yielded an expected NPV ranging from 13.1 to 13.4 Million EUR (approximately 30% lower than the deterministic solution considering RCP 2.6 climate trajectory), depending on the assumed dispersal capacity. The inclusion of the various sources of uncertainty decreased the loss in the objective function compared to the free scenario, with reductions to the VaR ranging from 1.2 to 3%.

### The risk of deterministic solutions

**Figure 5:**
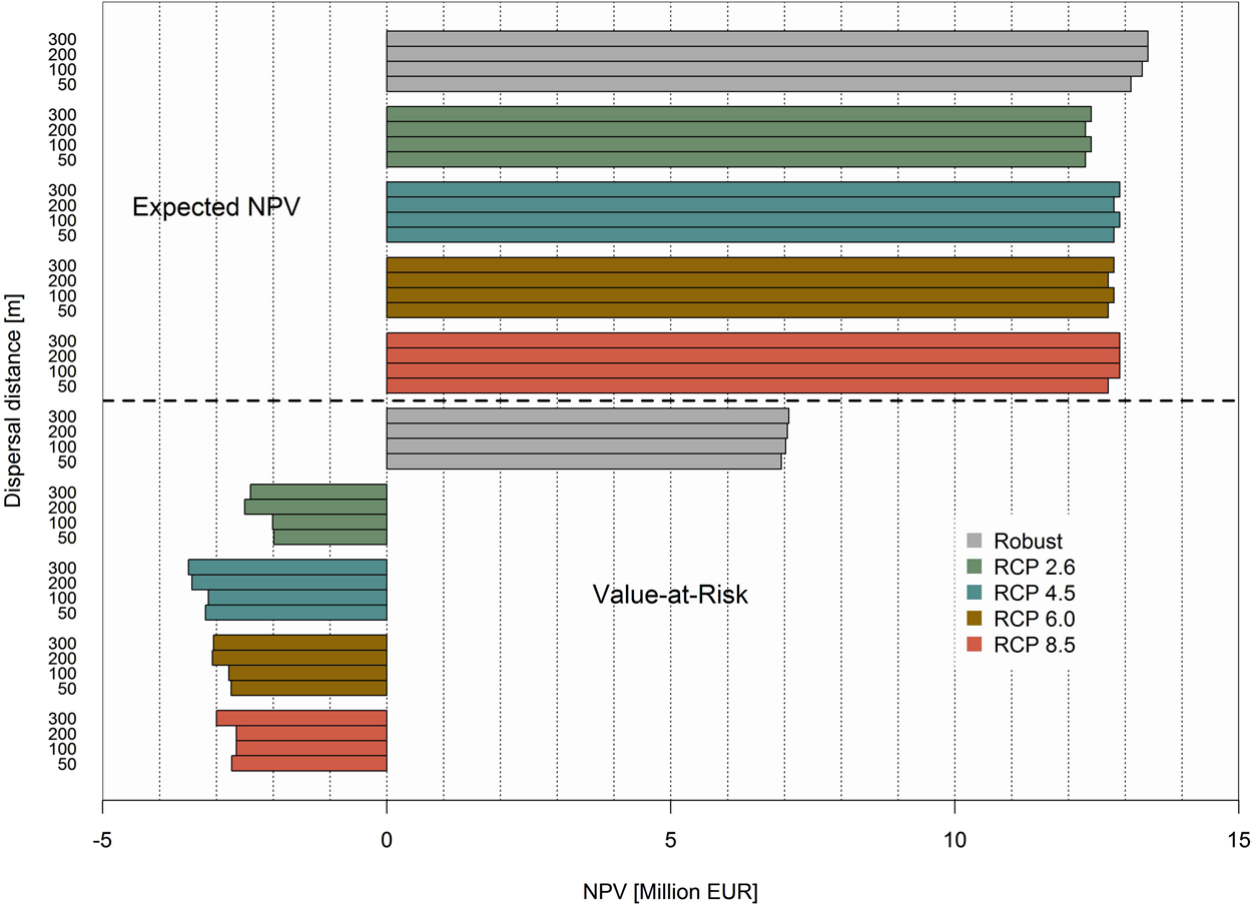
Performance of the robust and deterministic solutions taking into account multiple sources of uncertainty (climate development, economic development and forest disturbances) and dispersal capacities. The upper half of the figure shows the expected Net Present Value (NPV) of the solutions and the lower half displays the Value-at-Risk of the same solutions. The figure was created using RStudio 1.1.456 (https://www.rstudio.com/).

We assessed the economic outcomes of robust solutions towards various sources of uncertainty affecting forest management. Since forest profitability was reduced under uncertainty, one open question is how the optimal solutions obtained in the deterministic case would perform under uncertain conditions. Figure 5 shows the economic outcomes in terms of the expected NPV and the Value-at-Risk (5% quantile of the NPV distribution) when multiple sources of uncertainty (climate development, economic development and forest disturbances) were taken into account. Compared to the robust solution, the expected NPV yielded by the optimal deterministic solutions represented a loss from 3 to 8%, depending on the climate trajectory. If we consider the deterministic outcomes, the expected NPV under uncertainty was roughly half of the value obtained at our best guess (deterministic conditions). The impacts on the VaR were substantially more severe, with a reduction ranging from 129 to 150% compared to the robust solution, a loss of nearly 10 million EUR, indicating that the deterministic responses yielded considerably riskier options. Moreover, we observed that the climate RCP 4.5, showed the poorest performance under uncertainty, especially due to the high NPV deviations, evidenced by the lowest VaR values. Despite the reduction in the expected NPV, due to a loss of productive area for shorter dispersal distances (e.g. 50 m), the creation of the habitat network did not modify the portfolio of management alternatives, and thus the NPV did not vary strongly for different dispersal distances.

## Discussion

### Optimal allocation of conservation areas under uncertainty

The creation of forest reserves has been an important policy for promoting biodiversity in managed forest landscapes. Although reserves may help to balance the intensive land use in the surrounding matrix, due to its static nature, we may incur in the risk of producing several isolated forest reserves within the landscape that may not be able to support ecological processes^35^. Therefore, we propose an approach for coupling the strategic planning of forest reserves with the operational planning of allocating deadwood islands in the landscape, aiming to connect them. Our results indicate that allocating the deadwood islands based on the shortest distance to the forest reserves may not provide the best solution, and the consideration of local forest characteristics is warranted. In this sense, coupling forest planning at different hierarchical levels showed a beneficial impact on the outcome of forest management, corroborating with other studies integrating operational aspects into strategic forest planning (e.g. Kirby *et al*.^36^). Moreover, the creation of deadwood islands may benefit other red-listed species, e.g. *Osmoderma eremita*, in landscapes where these are present through the increased amount of habitat trees. Apart from providing habitat for saproxylic beetles, and consequently other saproxylic organisms, the creation of deadwood islands and forest reserves may also improve the habitat for other endangered taxa. For example, the increased number of veteran trees in the landscape may be especially beneficial for forest birds and bats^37,38^.

We perceived a significant modification in the habitat network configuration under uncertainty, as the profitability across the landscape varied substantially, leading to an economic infeasibility of several stands (negative NPV), thus allocating the whole area as deadwood islands. Here, we focused on the economic impacts of uncertainty for the habitat network selection, as a way to reduce the trade-off between production and conservation objectives. Nevertheless, besides the evident impacts on forest profitability, climate change is likely to alter ecological processes and community dynamics as well^39^. Thus, we may expect that for some taxa, especially those dependent on specific tree species, the creation of static forest reserves may not suffice, and additional conservation measures, such as the dynamic allocation of forest reserves or species translocation might be necessary in order to maintain communities at desirable levels^40,41^. These requirements may be easily adapted to our framework, by dynamically allowing the creation of new reserves and islands along the planning horizon.

We may further improve the efficiency of our conservation actions in the future, by finding areas with simultaneously high ecological and low economic value. In order to identify such critical areas, the coupling of forest growth models with models describing population dynamics and biological responses to climate change may provide insights on the most suitable conservation actions through the identification of thresholds and species responses under climate change, anticipating biodiversity losses^42^. Therewith, we could focus conservation efforts on target species with higher risk and apply suitable conservation actions in a timely manner.

### Optimal management portfolio and wood production

The management portfolio for the deterministic solutions favored a balance of selection forest systems both under the deterministic and robust approach, with an increase in early harvestings for the robust case. This combination of management alternatives enabled to anticipate revenues by harvesting stands with low growth rates and substitute them with faster growing stands, while applying low intensity harvesting alternatives for stands with higher growth rates, allowing them to grow at a faster rate than the interest rate applied. Apart from the possibility of early revenues, another reason for the promotion of selection forest systems was the lower cost related to the maintenance of habitat trees, due to the low value of the trees at the end of the simulation period. Although crop tree management regimes may provide candidate habitat trees with desirable characteristics, the complexity in forest structure provided by selection forests is also beneficial for conservation purposes^43^.

The management portfolio of the robust solution was substantially more diversified than the management portfolio of deterministic solutions, corroborated by other studies in the literature (e.g. Knoke *et al*.^44^). By diversifying the management portfolio and selecting management regimes with lower risk, it is possible to reduce the risk added by the covariance between stands and risky management options. Moreover, we observed an increase in the share of early harvestings in the robust case. This behavior arose from the fact that under uncertain conditions, especially under price fluctuations and disturbances, the risk of forest management increases due to the increased exposure to economic losses and it is advantageous to anticipate revenues. Amacher *et al*.^29^ point to the same patterns studying optimal rotation periods by incorporating storm risk into the Faustmann formula, indicating that the optimal rotation age reduces in the presence of stochastic disturbances, as the exposure to losses increases over longer periods. Additionally, taking into account the occurrence of storm damage, the increase in standing stock over time leads to an increased risk of economic losses in case a disturbance occurs. Therefore, an increase in early harvestings decreases the risk. The same authors point out that a similar behavior is expected under uncertain wood prices, as forest owners tend to reduce their risk by harvesting more today with price certainty rather than in the future with uncertain prices.

### Economic impacts of habitat selection and the role of uncertainty

The creation of the habitat network reduced the total forest NPV from 3.9 to 6.2%, compared to the baseline scenario, without the creation of new forest reserves and deadwood islands. These figures are typical for spatial optimization problems, especially the ones addressing adjacency constraints and connectivity of forest patches (e.g. Tóth and McDill^45^ and Öhman and Eriksson^46^). Moreover, we found an important impact of climate change in the outcomes of the optimization model, as the changes in forest productivity, especially the higher growth rates, increased the opportunity costs for the creation of the habitat network by 25%. Thus, indicating that climate impacts on productivity must be taken into account when deciding upon management activities.

We achieved suitable outcomes for the robust optimization model through the safe tractable approximation framework, allowing us to select a portfolio of management regimes with lower risk and higher expected NPV in face of climate, disturbance and economic uncertainty. Our results indicate that the performance of deterministic solutions suffer severely under uncertain conditions, thus highlighting the caveats of assuming deterministic conditions in the planning process, and the need for suitable planning tools for handling uncertainty. This phenomenon is well described in the literature. For example, Ben-Tal and Nemirovski^47^ investigated 90 linear optimization problems from NETLIB library, and found that for small data perturbations (0.1%) several problems became heavily infeasible.

The framework provided by robust optimization is arguably the most suitable tool for handling uncertainty in forest and conservation planning, as it allows for the consideration of only partial information on the distribution of data perturbations and accurate information on the distribution of the various sources of uncertainty affecting forest management is rarely available. Nevertheless, the robust counterpart of a deterministic problem usually becomes substantially more complex than the deterministic version. This is a strong limitation for large Mixed Integer Linear Programming (MILP) optimization problems, such as our harvest scheduling and habitat selection. One option for formulating the robust counterparts of MILP problems is the budgeted uncertainty approach^48^, which was also adapted for Linear Programming problems and applied to environmental planning problems (e.g. Knoke *et al*.^49^ and Messerer *et al*.^50^). This approach has the caveats of assuming that perturbations are independent and it may be computationally demanding for large instances of MILP problems. In this sense, the framework provided by Düzgun and Thiele^34^ seamlessly allows for coping with these issues by providing an efficiently computable formulation and including the covariance matrix characteristics into the problem formulation.

Despite the suitability of robust optimization (RO) techniques for handling uncertainty in forest and conservation planning, the applications are still scarce (e.g. Messerer *et al*.^50^; Kašpar *et al*.^51^; Palma and Nelson^52^). Hence, a further investigation of RO tools for conservation planning is warranted, especially when considering changing environmental conditions. Climate change adds deep uncertainty to forest management and non-stochastic approaches, such as RO and robust decision making (RDM), allow for the consideration of such deep uncertainty into the planning process, providing solutions that perform well over a wide range of possible future scenarios^53–55^. Therewith, managers may apply these frameworks to select a safe course of action, anticipating possible climate impacts on forest ecosystems and guaranteeing minimum levels for the provisioning of ecosystem goods and services, according to their objectives and preferences.

### Limitations

We did not account in our analysis for changes in species distribution and preferences for specific tree species, since the latter requires a long-term adaptation action and a longer period than the one applied in this study. Nevertheless, due to changing climatic conditions, habitat suitability and species distribution may change (e.g. Hanewinkel *et al*.^25^ and Zhu *et al*.^56^). Therefore, coupling a species distribution model with the forest growth model may be applied in order to predict habitat suitability under environmental changes and perform the conservation planning accordingly, anticipating possible changes in habitat availability. Similarly, an evaluation of the dynamics of disturbance regimes under climate change is warranted, since novel disturbances may appear in areas where they did not previously occur (e.g. forest fires caused by extreme drought events). Moreover, for the creation of forest reserves we consider current policy pledges to extend the share of set aside areas in Germany to 10%. A further investigation of the impacts of modifying this requirement can provide useful information on the trade-offs of increasing the share of forest reserves under uncertainty.

The consideration of model and parametric model uncertainty, not accounted for in our study, may exert influence on the selection of robust management alternatives in the future (e.g. Augustynczik *et al*.^57^). Therefore, an extension of our models to include other possible relevant sources of uncertainty is recommended in order to provide a more complete risk analysis.

We did not include effects of climate change on the probability of storm occurrence, since the interactions between climate change and maximum wind speed behavior is still inconclusive^4^. However, this behavior might differ regionally and wind speeds might increase under climate change (e.g. Andersson *et al*.^58^). The implementation of wind speed patterns may be included in our framework, by an updating of the Gumbel distribution parameters, describing the probability of maximum wind speeds. One must also consider that the application probabilistic and mechanistic disturbance models may result in contrasting recommendations. Usually, for probabilistic models early harvestings and decrease in standing stock will result in lower risk, whereas the same interventions may increase canopy roughness and the risk of wind damage predicted by mechanistic models^59^. Moreover, our analysis also did not include other important forest disturbances in the study area, such as bark beetle outbreaks and snow damage, that may further reduce the expect NPV and VaR obtained.

We considered that management regimes were independent in our study, thus leading to a diversification of the management portfolio. In this sense, the risk of the management portfolio may be underestimated if in reality these conditions do not hold. Thus, the covariance between stands under different management regimes can be included in the same framework when the size of the problem is not computationally prohibitive.

## Conclusion

Our study shows that forest disturbances, climate development, dispersal capacity of the saproxylic organisms and economic conditions exert strong influence on the outcomes of forest management and the operationalization of conservation actions. Still, forest and conservation planning has been traditionally performed assuming perfect knowledge regarding future conditions and disregarding uncertainty. Here we demonstrated that the consideration of uncertainty modifies substantially the optimal allocation of deadwood islands and forest reserves. The performance of deterministic solutions suffers severely under uncertainty and the corresponding risk can be substantially higher. Hence, assuming such deterministic conditions may lead to wrong indications of future forest profitability and poor information for the design of the habitat network and for adaptive management actions. In this sense, robust optimization tools seamlessly allow to include deep uncertainty introduced into the decision-making process and enable decision-makers to select now a safe course of action and reduce the risk of adverse outcomes in the future.

## Methods

### Forest simulation

In order to simulate the forest development under climate change, we applied the single-tree, distance dependent growth model Sibyla. We simulated 50 alternative management regimes under four different climate change trajectories applying the Global Circulation Model HadGEM2-ES and bias corrected by ISI-MIP (details in Supplementary 3).

### Economic evaluation of management regimes

For the economic evaluation of the different management regimes, we applied the wood prices for each assortment class used in Baden-Württemberg in 2016, and harvesting costs according to Härtl *et al*.^60^. The profitability of each management regime was evaluated in terms of the net present value (NPV) generated during the simulation period:

1 $$NPV=-\,IniStock+\sum _{i\in T}\frac{ThinNe{t}_{i}}{{(1+r)}^{i-1}}+\frac{FinStock}{{(1+r)}^{T-1}}$$

where: *IniStock*: value of standing stock at the beginning of the simulation; T: simulation period; *ThinNet*_*i*_: net thinning revenue in year i; r: discount rate; *FinStock*: value of the standing stock at the end of the simulation period.

The NPV was computed for each management regime i, as the sum of the net thinning revenues and the value of the standing stock at the end of the simulation period minus the value of the standing stock at the beginning of the simulation period, defined as the initial investment (Eq. 1). Additionally, we corrected the obtained NPV for each management regime according to the retention forestry practices recommended in the region of the study area^61^. To this end, in a post-simulation analysis, we reduced the obtained NPV by the value of habitat trees and deadwood left on the stand. For habitat trees, we considered that 2.5 to 5 crop-trees/ha are left on the stand as habitat trees, leaving 2.5 habitat trees /ha for spruce-dominated stands and 5 habitat trees/ha for other species. The reduced number of habitat trees for spruce stands has the objective of mitigating the risk of bark-beetle outbreaks, an important disturbance factor in the region^62^. Moreover, we assumed a deadwood volume target of 35 m³/ha, following the recommendation for mixed-montane forests provided by Müller and Bütler^63^ as a threshold for sustaining saproxylic beetles on forest stands. Taking into account the input necessary for sustaining the deadwood volume above this threshold (0.9 m³/ha/year), we supplemented the necessary deadwood amount with thinned trees if the volume of dead trees was below this value and in case the deadwood volume was above this minimum input, we considered as a thinning revenue with reduced price.

### Sources of uncertainty

#### Uncertainty in climate development

Taking into account the multiple effects of climate change over forest ecosystems, including possible effects of climate change is key for forest planning, in order to anticipate possible changes in forest productivity and adapt forest management accordingly. The most challenging aspect of climate change for decision-making, however, lies on the fact that we cannot identify a single scenario to predict climate development and thus we must consider a set of plausible climate change scenarios^53^. In this context, to include climate change uncertainty in our planning problem, we assessed the model outputs for each management regime under four different climate change trajectories. These trajectories were defined based on a combination of the Global Circulation Model HadGEM2-ES under the Representative Concentration Pathways (RCP) 2.5, 4.5, 6.0 and 8.5, bias corrected by ISI-MIP (Table 1). The RCPs represent a set of possible futures for atmospheric composition, where RCP 2.6 represents a strong mitigation scenario, RCP4.5 and RCP6.0 represent medium mitigation scenarios with emissions peaking around 2040 and 2080 respectively, and RCP8.5 a high emission scenario^64^. We derived for each climate trajectory the corresponding climatic variables used as input in Sibyla (vegetation length, average temperature during the vegetation period, precipitation during the vegetation period, temperature range and atmospheric CO_2_ and N_2_O concentrations) and simulated the 50 management regimes under each of the four climate change trajectories.

**Table 1:** The table describes the four climate change trajectories considered in our analysis as a combination of a Global Circulation Model (GCM) and a Representative Concentration Pathway (RCP).

Trajectory	GCM	RCP
1	HadGEM2-ES	2.6
2	HadGEM2-ES	4.5
3	HadGEM2-ES	6.0
4	HadGEM2-ES	8.5

#### Uncertainty in economic conditions

We considered two main sources of uncertainty in economic conditions affecting directly forest profitability, namely the uncertainty in wood price development and the uncertainty in discount rates. For the wood price development, we generated for each management regime and climate change trajectory, 100 price deviates according to a Geometric Brownian Motion (GBM). GBM processes are well suited for describing wood price development^65^ and can be described as:

2 $${P}_{n}={P}_{0}{e}^{X(t)}$$

where: *P*_*n*_ price at period n; *P*_0_: initial price; *X*(*t*): Brownian Motion.

We simulated the 100 price developments for each species and assortment class, by applying as initial price the wood prices used in Baden-Württemberg in 2016. For computing the Brownian Motion (*X*(*t*)), we used yearly price data from the period 2000 to 2016, corrected by inflation rates, to calculate yearly percentage changes in wood prices. We then generated for each of the 100 simulations, 50 random deviates of the corresponding GBM, ignoring the drift, thus establishing 100 price developments during our simulation period of 50 years. We obtained unrealistic price developments for the smallest assortment class (energy and pulpwood), especially for beech and oak, due to the long simulation period and the high volatility of this assortment during the past decade. Hence, it was necessary to apply the volatilities of the neighboring assortment class in order to maintain prices within reasonable ranges.

We considered different discounting schemes that may be applied by forest managers according their risk perception. To this end, in combination with price uncertainty, for each of the 100 NPV evaluations we considered a random deviate of discounting rates. We considered a risk-free discount rate of 0.67% and summed a random deviate from an exponential distribution with rate parameter equal to 3.5, resulting in a decreased probability as interest rates increased^66^. Therewith, we mapped the discount rate from [0.067, 0.03], considering the 99.9% quantile of the exponential distribution.

#### Uncertainty in forest disturbances

Wind is the most important disturbance agent in the study region and has resulted in significant losses to the forest sector during the past decades^62,67^. Therefore, we also considered the uncertainty in NPV caused by wind damage risk in our analysis, in a similar fashion as applied to economic uncertainty. We assessed the wind risk with the help of the model proposed by Schmidt *et al*.^68^:

3 $$\begin{array}{rcl}g({\pi }_{i}) & = & {\beta }_{1i}+\,\mathrm{log}(\frac{DB{H}^{\alpha i}}{{h}^{-\gamma i}})+{\beta }_{2i}Top\_to\_dist1+{\beta }_{3i}Top\_to\_dist2\\  &  & +{\beta }_{4i}Top\_to\_dist3+{\beta }_{5i}Top\_to\_dist4+f(N,E)\end{array}$$

(Adapted from Schmidt *et al*.^68^).

where: *g*(*π*_*i*_): logit link function of the damage probability of a given tree of species i; *β*_1*i*_ *to β*_5*i*_: species-specific model parameters; *f*(*N*,*E*): smoothing function according to tree coordinates (Northing and Easting).

The probability of tree damage (Eq. 3) is assessed based on the tree diameter at breast height (DBH), height (h), the topex-to-distance index (top_to_dist) and the tree coordinates. The topex-to-distance is a measure of exposure according to the topographic characteristics of the site to the relevant wind directions during the storm, expressed as a sum of the angles of the terrain slopes. The smoothing function corrects the damage probability according to tree coordinates taking into account the coordinates of the storm center (establishing a proxy for the wind speed) and the h/DBH ratio is a proxy for tree stability (for details see Schmidt *et al*.^68^).

As the model was parametrized for a single storm, to enable the risk assessment related to wind damage, we evaluated the occurrence probability of a comparable storm, in terms of wind speed. We applied a similar approach used in the mechanistic wind damage model ForestGALES^69^, in which the annual occurrence probability of a given wind speed is estimated with the help of a Gumbel distribution (Generalized Extreme Value distribution Type-I):

4 $$AOP=1-{e}^{-{e}^{-(x-\mu )/s}}$$

where: *AOP*: annual occurrence probability; *x*: wind speed; *μ*: location parameter; *s*: scale parameter.

We used the hourly data recorded by the nearest weather station to our research area (Feldberg weather station) to fit the Gumbel distribution parameters. To this end, we applied the R package fitdistrplus^70^ to the maximum yearly wind speed occurrences from the period 1969 to 2016. Taking into account the wind speed of 41.67 m/s recorded in the same weather station during the storm “Lothar”^71^ we defined the annual occurrence probability of a same wind speed. Subsequently, at each of the 100 NPV calculations, and each simulation year, we generated a random uniform deviate, considering that the storm would occur if the random deviate was smaller than the respective annual occurrence probability. Therewith, in case the storm occurred during the current NPV evaluation, we calculated the damage probability based on the average tree on the stand. For each species in each stand we used this probability of damage as a proxy of the amount of damage. Hence, if the probability of damage of the average tree was equal to 0.3, we assumed that 30% of the stand was damaged. Accordingly, we inputted the damaged volume into the deadwood pool, and reduced the harvested volume and standing volume by the same proportion in the periods after the occurrence of the storm. In addition, we established that in the periods in which the storm occurred, the wood price was reduced by 25%, as a result of the oversupply of wood, consistent with the observed price reduction after the storm Lothar^72^.

### Optimization models

#### Deterministic optimization model

Our optimization problem had the objective of maximizing forest NPV, while creating new forest reserves, amounting to 10% of the total area and connecting these forest reserves with a network of deadwood islands. For ensuring that forest reserves respect a minimum area, we applied the ring-inequalities^73^ and for connecting them we applied an adaptation of the Single Commodity flow problem (see Minoux *et al*.^74^). The optimization model is as follows:

5 $$MaxZ=\sum _{i\in S}\sum _{j\in M}np{v}_{ij}{x}_{ij}-\frac{np{v}_{ij}}{are{a}_{i}}aux{z}_{ij}$$

Subject To

6 $$aux{z}_{ij}\le BPT{x}_{ij}\,\forall \,i\in S,\,\forall \,j\in M$$

7 $$aux{z}_{ij}\ge {z}_{i}-BPT(1-{x}_{ij})\,\forall \,i\in S,\,\forall \,j\in M$$

8 $$aux{z}_{ij}\le {z}_{i}\,\forall \,i\in S$$

9 $$\sum _{j\in M}{x}_{ij}+{y}_{i}=1\,\forall \,i$$

10 $$\sum _{i\in S}\sum _{j\in M}{v}_{ijk}{x}_{ij}\ge 0.7VolB\,\forall \,k\in P$$

11 $$\sum _{i\in S}\sum _{j\in M}{v}_{ijk}{x}_{ij}\le 1.3VolB\,\forall \,k\in P$$

12 $$\sum _{i\in S}\sum _{j\in M}ini{v}_{ij}{x}_{ij}\le \sum _{i\in S}\sum _{j\in M}fin{v}_{ij}{x}_{ij}$$

13 $$\sum _{u\in \partial C}{y}_{u}\ge \sum _{i\in C}{y}_{i}-(|C|-1)\,\forall \,C\subset {C}_{MA}$$

14 $$\sum _{i\in S}are{a}_{i}{y}_{i} > 0.1Tarea$$

15 $$\sum _{i\in PT}p{t}_{i}=sumPT$$

16 $$\sum _{i\in {G}_{j}}p{t}_{i}={z}_{j}\,\forall \,j\in S$$

17 $${z}_{i}\ge {y}_{i}\,\forall \,i\in S$$

18 $$linSu{m}_{ij}\le |PT|{k}_{ij}\,\forall \,(i,\,j)\in AdjPT$$

19 $$linSu{m}_{ij}\le sumPT\,\forall \,(i,j)\in AdjPT$$

20 $$linSu{m}_{ij}\ge (1-{k}_{ij})|PT|\,\forall \,(i,\,j)\in AdjPT$$

21 $$p{t}_{i}\ge {k}_{ij}\,\forall \,(i,\,j)\in AdjPT$$

22 $$p{t}_{j}\ge {k}_{ij}\,\forall \,(i,\,j)\in AdjPT$$

23 $$\sum _{(ROOT,i)\in AdjPT}flo{w}_{ROOTi}=sumPT-1$$

24 $$flo{w}_{ij} < linSu{m}_{ij}\,\forall \,(i,\,j)\in AdjPT$$

25 $$flo{w}_{ij}=flo{w}_{jk}-1\,\forall \,(i,\,j)\in AdjPT$$

26 $${x}_{ij},\,aux{z}_{i},\,p{t}_{i},\,linSu{m}_{ij},\,{k}_{ij}\in \{0,1\}$$

The objective function (Eq. 5) maximizes forest NPV while applying a correction by reducing it according to the area selected as deadwood islands. Constraints (Eq. 6) to (Eq. 8) enforce the auxiliary variable *auxz*_*ij*_ to assume the value of the area of stand i selected as deadwood island case management j is selected and value 0 otherwise. Constraint (Eq. 9) imposes that stands are either managed or forest reserves. Constraints (Eq. 10) and (Eq. 11) are wood flow constraints and guarantee that the wood produced every period does not vary more than 30% compared to the volume bound. Constraint (Eq. 12) guarantees that the standing volume at the end of the simulation period is greater or equal to the standing volume at the beginning of the simulation period. The set of constraints (Eq. 13) are the ring-inequality constraints and ensure that groups of stands selected as forest reserves respect a minimum area. It enforces that if a group of stands is selected as a forest reserve and its combined area is inferior to minimum area limit, at least one neighbor stand to the group is also selected as a forest reserve. Constraint (Eq. 14) ensures that at least 10% of the total forest area is set aside as forest reserves. Constraint (Eq. 15) assign the total number of deadwood islands in the solution to the variable *sumPT*. Constraints (Eq. 16) links the number of deadwood islands within a given stand i with the variable *z*_*i*_. Constraint (Eq. 17) guarantees that each forest reserve contain at least one deadwood island, thus ensuring that all forest reserves to be connected by a network of deadwood islands. Constraints (Eq. 18) to (Eq. 20) represent a linearization of the multiplication of the variables *k*_*ij*_ by variable *sumPT*. Constraints (Eq. 21) and (Eq. 22) ensure that if an arc *k*_*ij*_ is part of the solution, the corresponding center points connected by this arc are also selected to be part of the solution. Constraint (Eq. 23) injects the appropriate amount of flow into the network, starting from the root point. (Eq. 24) bounds the amount of flow traveling through an arc to the number of points in the network. Constraint (Eq. 25) represents the set of flow conservation constraints, stating that at each center point, the ingoing flow is equal to outgoing flow minus one, i.e. one unit of flow is consumed at each center point. Constraint (Eq. 26) enforces that variables assume binary values. The description of variables, sets and data is provided in Table 2.

**Table 2:** List of the sets, variables and input data applied in the optimization models.

Set	Description
S:	set of stands in the area
M:	set of management regimes
*P*:	number of periods
*C*:	block of stands that do not respect the minimum area limit
∂*C*:	neighborhood set of the block C
*C*_*MA*_:	set of all blocks of stands that do not respect the minimum area limit
*G*_*i*_:	set of center points within stand i
*PT*:	set of all candidate center points for deadwood islands
**Function**	**Description**
*L*(Φ(*x*))	Piecewise linearization of the function In(cosh(*x*))
**Variable**	**Description**
*x*_*ij*_:	binary decision variable that takes value 1 case stand i is managed under regime j or 0 otherwise
*auxz*_*i*_	integer auxiliary variable that equal the variable z_*i*_ case variable *x*_*ij*_ takes value 1 and 0 otherwise
*z*_*i*_:	integer variable that equal the number of deadwood islands allocated to stand i
*y*_*i*_:	binary variable that takes value 1 case stand i is selected as forest reserve and value 0 otherwise
*VolB*:	bound for the volume production at each period k
*pt*_*i*_:	binary variable that takes value 1 case center point i is selected as part of the solution and value 0 otherwise
*sumPT*:	variable that expresses the total number of points in the deadwood island network
*linSum*_*ij*_:	auxiliary variable that linearizes the multiplication of the variable *sumPT* by variables *k*_*ij*_
*k*_*ij*_:	binary variable that assumes value 1 case the arc connecting center points i and j is selected to be part of the solution and value 0 otherwise
*flow*_*ij*_:	flow travelling through arc (i,j)
**Data**	**Description**
*npv*_*ij*_:	Net Present Value generated by stand i under management regime j
*area*_*i*_:	area of stand i
*Tarea*:	total forest area
$|C|$:	number of stands in block C
*BPT*:	bound on the number of center points in a single stand
*AdjPT*:	adjacency matrix of the set of points *PT*
$|PT|$:	number of points in *PT*
*v*_*ijk*_:	volume produced by stand i, under management j in period k
**E***npv*:	Expected Net Present value
*chol*_*ik*_:	i-th row and k-th column element of the Cholesky decomposition of the covariance matrix

#### Robust counterpart applying a safe tractable approximation

For the robust counterpart of the deterministic problem, we applied a safe tractable approximation approach, based on the framework proposed by Düzgun and Thiele^34^ (see details in Supplementary 4):

27 $$MaxZ=\sum _{i\in S}\sum _{j\in M}({\rm{E}}np{v}_{ij}{x}_{ij}-\frac{{\rm{E}}np{v}_{ij}}{are{a}_{i}}aux{z}_{i})-t$$

28 $$-t+\beta \sum _{j\in M}\sum _{i\in S}(L({\rm{\Phi }}(\frac{\sum _{k\in S}cho{l}_{jki}{x}_{kj}}{\beta })))-\beta \sum _{j\in M}\sum _{i\in S}(L({\rm{\Phi }}(\frac{\sum _{k\in S}cho{l}_{jki}\frac{aux{z}_{kj}}{are{a}_{k}}}{\beta })))+\beta \,\mathrm{ln}(\frac{1}{0.05})\le 0$$

For the robust counterpart of our planning problem, the objective function of the deterministic problem (Eq. 5) was replaced by the objective function (Eq. 27) and constraint (Eq. 28) was added. Constraint (Eq. 28) computes the 95%quantile of the NPV deviation based on a Bernstein approximation scheme, reducing the expected total NPV value by the same amount. During the computation of the Cholesky decomposition of the covariance matrix, we ran into numerical problems, due to occurrence of negative eigenvalues in the covariance matrix. In order to solve this issue, we cleaned the covariance matrix by computing the closest positive definite matrix applying the R package “corpcor”^75^. For obtaining an estimate of the *β* value, we solved the non-linear form of (Eq. 28), minimizing the value of t and restricting the solution to be equal to the deterministic optimal solution, establishing a proxy for *β*. We then analyzed different *β* values close to the proxy value, evaluating the obtained solutions and choosing the best one.

For the selection of the deadwood islands network, we created a grid of candidate center points in the software ArcMap 10.1 (ESRI), spaced 60 by 60 m from each other. We considered 1 ha circular shaped deadwood islands, with 56.4 m radius. Furthermore, we defined that two deadwood islands may not be separated by a distance superior to the dispersal capacity of saproxylic beetles. We used the saproxylic beetles *Lucanus cervus* as indicator species. Deadwood islands are more likely to provide habitat for these species, compared to manage forests, as it requires large amounts of deadwood. The reported typical dispersal range for *L. cervus* varies from 50 to 700 m, similar to ranges found by other indicator species, such as *Osmoderma eremita* (50 and 1500 m) with decreasing probability as distance increases^32,33,76^. We highlight that our approach may be seamlessly adjusted to the dispersal of different indicator species and taxa using the same resources, namely deadwood and habitat trees. We constructed the adjacency matrix applying an dispersal distances of 50, 100, 200 and 300 m by considering any two center points with Euclidean distance inferior to 162.8 to 412.8 m (dispersal distance plus two times the deadwood island radius) as adjacent. For the minimum area constraints, we used a 50 ha limit. Moreover, the candidate set of stands for the creation of new forest reserves was restricted to beech or silver fir-dominated stands with age superior to 160 years, according to recommendations from the Forest Administration in the state of Baden-Württemberg^61^.

We applied a 1% discount rate for the NPV evaluation in the deterministic case, according to the long-term interest rate reported by the European Central Bank in Germany during the last 5 years (ECB), whereas for the robust case, all sources of uncertainty were simultaneously considered for the analysis of forest NPV. We solved the optimization models using Gurobi 7.5.2^77^, accepting solutions with an optimization gap of 3% for the deterministic case and 5% for the robust case, compared to the upper bound on the optimal solution.

## Electronic supplementary material

Supplementary Information

## Data Availability

The authors confirm that all the relevant data are available through the lead author.
