# Supplementary material for: Multiple uncertainties require a change of conservation practices for saproxylic beetles in managed temperate forests
Source: Sci Rep. 2018 Oct 8;8:14964. doi: 10.1038/s41598-018-33389-9 (PMC6175923; doi:10.1038/s41598-018-33389-9)
Supplement: Supplementary file 1 — Supplementary Information [file 41598_2018_33389_MOESM1_ESM.docx]

Title page

Multiple uncertainties require a change of conservation practices for saproxylic beetles in managed temperate forests

Andrey L. D. Augustynczik*^1^, Rasoul Yousefpour^1^ & Marc Hanewinkel^1^

*Corresponding author ([andrey.lessa@ife.uni-freiburg.de](mailto:andrey.lessa@ife.uni-freiburg.de))

^1^Chair of Forestry Economics and Forest Planning, University of Freiburg, Tennenbacher Str. 4 (2. OG)

D-79106 Freiburg

Supplementary 1 – Study area

1 Study area

Our study area is a mixed-montane forest located between the coordinates 47°56’30’’ and 47°51’36’’N and 7°58’12’’ and 8°6’36’’ E, in the Southern Black Forest. The area is a state forest composed by 582 stands, with a total area of 2679 ha (Figure 1), representing a typical management area of a forest enterprise, in terms of extent and species composition. The soils are predominantly brown earth, with soil moisture class fresh to moderately dry and the climate is classified as Cfb according to Köppen. The stands are predominantly located in montane (450-800m) and high montane (800-1500m) altitudinal belts.

The managed area amounts to 2534 ha (94.5%), whereas 145 ha (5.5%) are forest reserves created in 1993 (banned forest “Scheibenfelsen”, in the north of the study area) and 1991 (banned forest “Feldseewald”, in the southern part of the study area). These areas are set aside from forest management, and interventions are only allowed if they risks of pest and diseases outbreaks for neighboring forests are present (Landesrecht BW 1995). The dominant species are *Picea abies* (62%), *Fagus sylvatica* (18%) and *Abies alba* (7%), and other species represent 13% of the forest cover, with low productive yield classes for broadleaves and intermediate to low productivity classes for conifers.


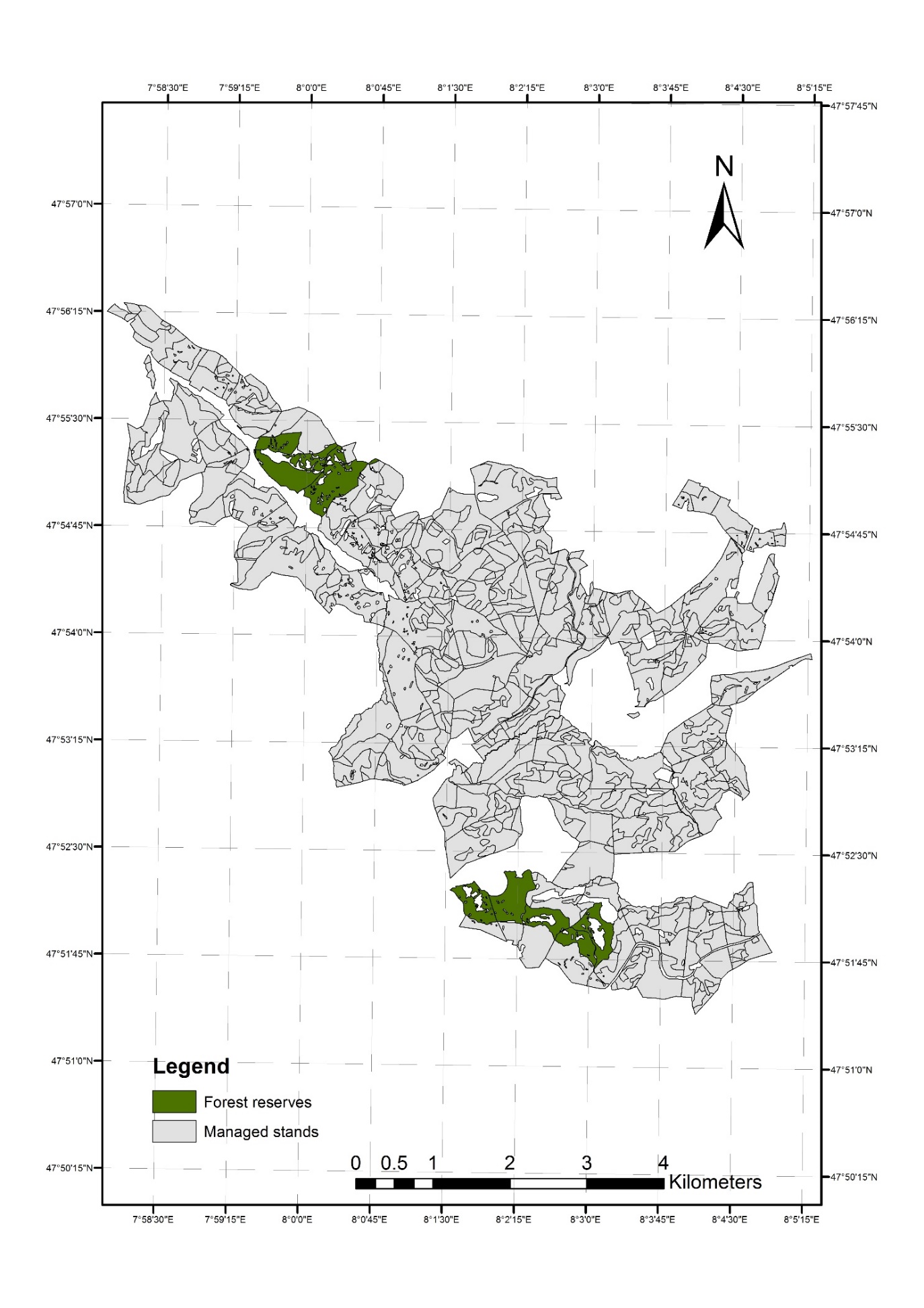


S1-Figure 1. Study area with forest stands and forest reserves highlighted. The map was created using ArcMap 10.3.1 (http://desktop.arcgis.com/en/arcmap/).

Title page

Multiple uncertainties require a change of conservation practices for saproxylic beetles in managed temperate forests

Andrey L. D. Augustynczik*^1^, Rasoul Yousefpour^1^ & Marc Hanewinkel^1^

*Corresponding author ([andrey.lessa@ife.uni-freiburg.de](mailto:andrey.lessa@ife.uni-freiburg.de))

^1^Chair of Forestry Economics and Forest Planning, University of Freiburg, Tennenbacher Str. 4 (2. OG)

D-79106 Freiburg

Supplementary 2 – Additional results of efficient allocation


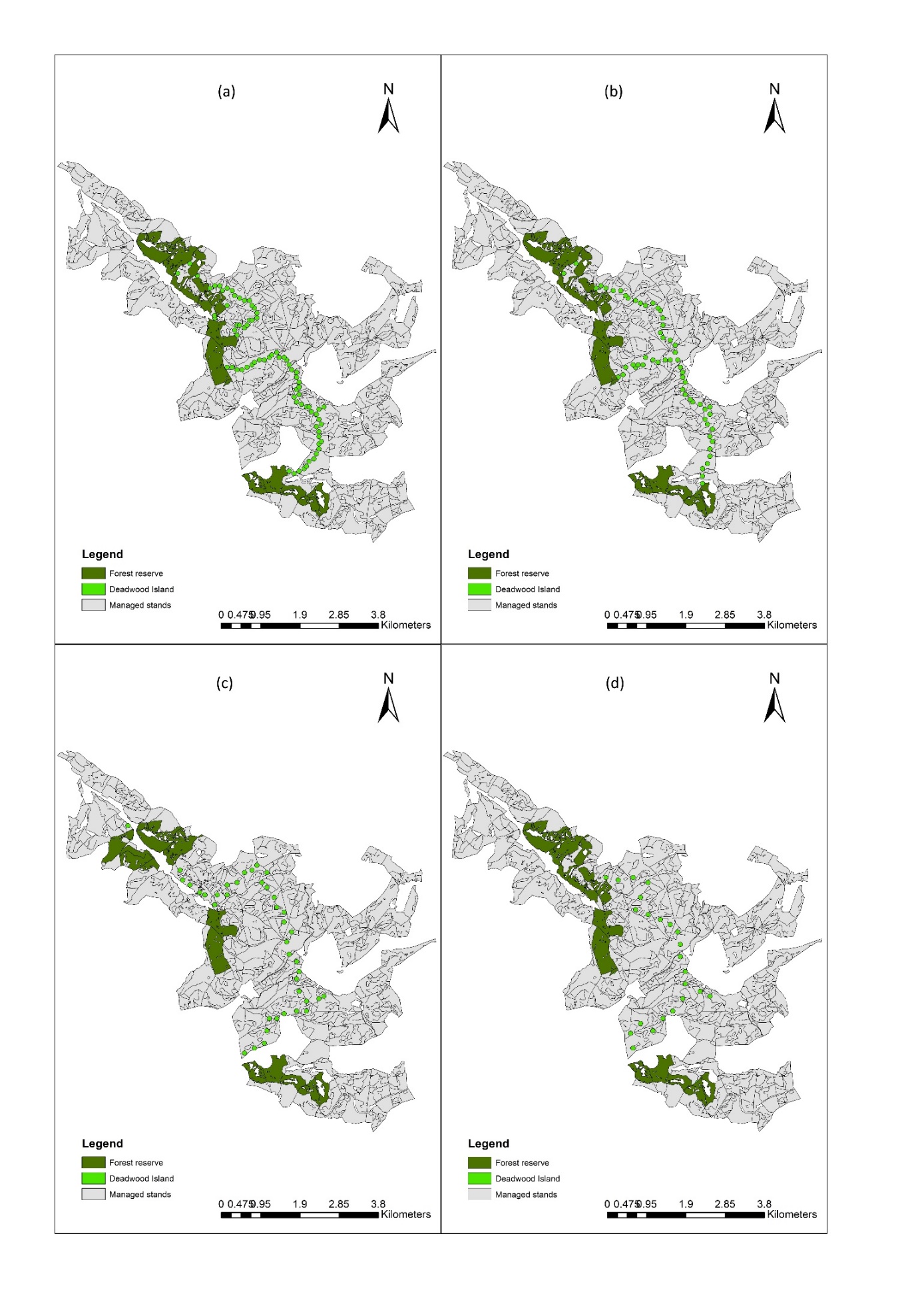


S2-Figure 1. The figure shows the optimal allocation of deadwood islands for the deterministic case, considering climate trajectory RCP 4.5 for a dispersal capacity of 50m (a), 100m (b), 200m (c) and 300m (d). The map was created using ArcMap 10.3.1 (http://desktop.arcgis.com/en/arcmap/).


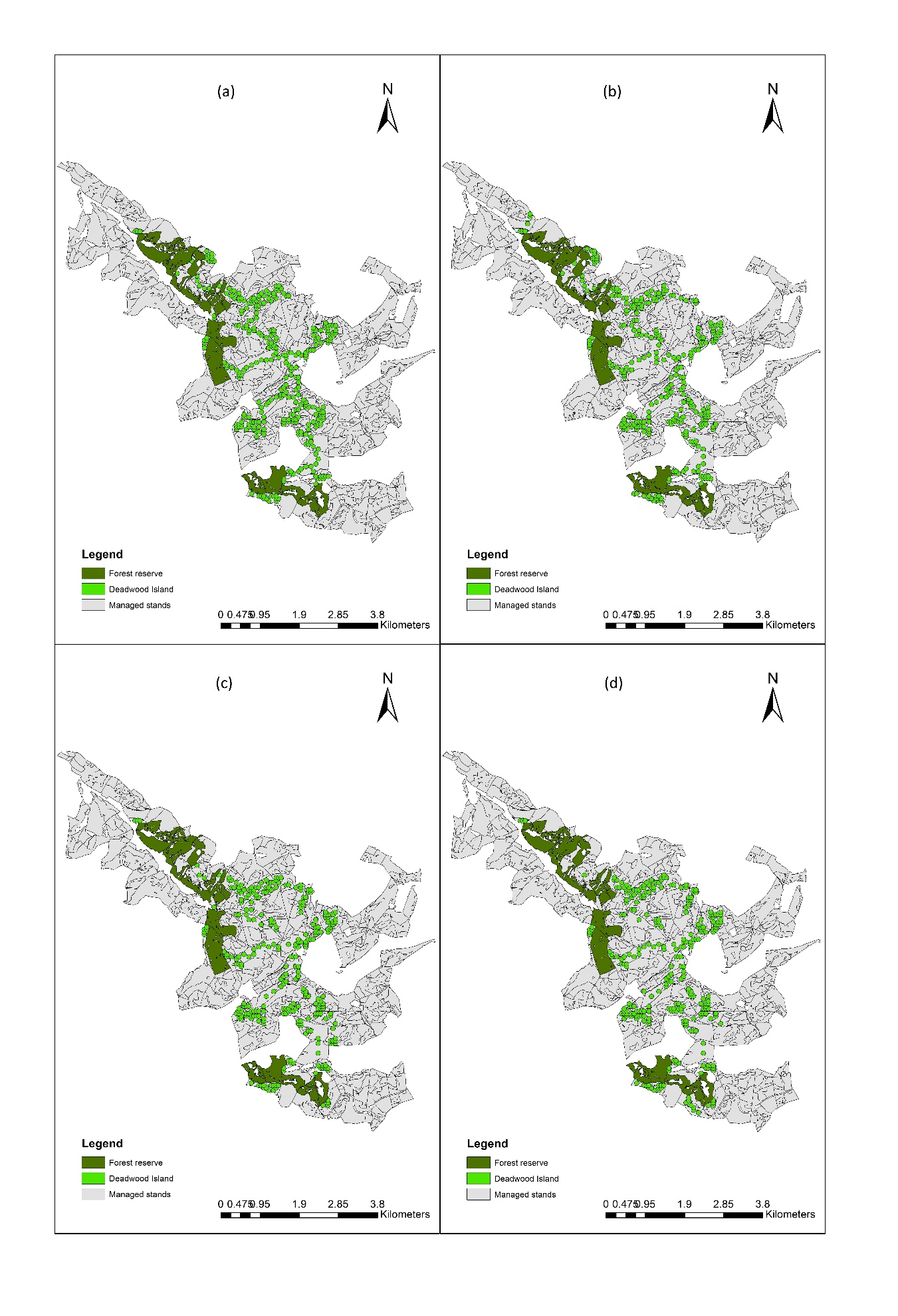


S2-Figure 2. The figure shows the optimal allocation of deadwood islands for the robust case, considering multiple sources of uncertainty for a dispersal capacity of 50m (a), 100m (b), 200m (c) and 300m (d). The map was created using ArcMap 10.3.1 (http://desktop.arcgis.com/en/arcmap/).

Title page

Multiple uncertainties require a change of conservation practices for saproxylic beetles in managed temperate forests

Andrey L. D. Augustynczik*^1^, Rasoul Yousefpour^1^ & Marc Hanewinkel^1^

*Corresponding author ([andrey.lessa@ife.uni-freiburg.de](mailto:andrey.lessa@ife.uni-freiburg.de))

^1^Chair of Forestry Economics and Forest Planning, University of Freiburg, Tennenbacher Str. 4 (2. OG)

D-79106 Freiburg

Supplementary 3 – Forest simulation and data

1 Forest simulation

The simulation of forest development was carried out applying the forest growth simulator Sibyla ^1^. Sybila is a semi-empirical distance-dependent individual tree model, thus simulating the growth and mortality of individual trees on forest stands ^2^. The model is climate sensitive and works based on potential height and diameter increments, which are then reduced to actual increments based on site conditions, tree vitality and competitive pressure ^3^. For the site reduction factor, numerous climatic and edaphic parameters are assessed, including the vegetation length, average temperature during the vegetation period, precipitation during the vegetation period, temperature range, soil water, nutrient supply and atmospheric CO_2_ and N_2_O concentration ^2^. This site reduction factor is then combined with a tree vitality index (based on the crown surface area) and a competition index ^4^, reducing the potential growth of height and diameter, yielding the actual increment ^2^. With the help of allometric equations, the increment is used to derive the stand structure in terms of wood volume, wood assortments and biomass of different tree compartments.

We simulated the forest development for a 50-years period, with management interventions every 10 years. The changes in development phase for the management definition were species-dependent, as well as the default crop tree release method and target DBH for diameter harvesting interventions, based on the dominant height of the stand (Table 1). To evaluate the timing of application of different thinning methods we simulated the development of each stand without thinning interventions and defined the appropriate measure for each species and each period. We established management alternatives defining a factorial combination of three options for the number of selected of crop-trees, three options for crop-tree releasing (in terms of number of competitors and clearing radius) and three options for target DBH, generating 27 alternatives in Table 2 (3 options for the crop tree number x 3 options for the release differentiated for conifers and broadleaves x 3 options for the target DBH). We tested 12 options of thinning from below schemes, applying different harvesting levels as a factorial combination, according to the three development phases of the stand (young, growing and utilization phases), including two options for thinning intensity for the young phase, three options for the growing phase and two options for the utilization phase, generating 12 management alternatives (Table 3). In addition, we designed 10 management alternatives for selection forest systems, by applying five options for the target DBH in the standard Liocourt function and the five types of selection forest (A to E) for the Meyer function (Eq.1 and Eq.2) (Table 4). The selection forest systems did not differentiate between development phases of the stand, since it creates a forest in steady state, with similar structure and harvesting levels along the rotation. The last management alternative applied no thinning interventions.

S3-Table 1. The table displays the management regime applied for each species, where hdom young is the dominant height (height of the 100 trees with largest DBH per ha) up to the species are in the young development phase and hdom growing is the dominant height up to the species are in the growing phase. Crop trees specify the number of selected crop trees per hectare for each species, amount shows the number of removed competitors for each conifer species and Radius show the clearing radius around broadleaf crop trees. Target DBH displays the target diameter for final harvesting.

| Species | hdom young (m) | hdom (m) growing | Crop trees (N/ha) | Amount (N/crop tree) | Radius (m) | Target DBH (cm) |
| --- | --- | --- | --- | --- | --- | --- |
| *Abies alba* | 16 | 28 | 150 | 2 | - | 50 |
| *Acer pseudoplatanus* | 14 | 30 | 50 | - | 4 to 9 | 50 |
| *Alnus glutinosa* | 14 | 30 | 50 | - | 4 to 9 | 50 |
| *Betula sc.* | 15 | 28 | 50 | - | 4 to 9 | 50 |
| *Castanea sativa* | 14 | 30 | 50 | - | 4 to 9 | 50 |
| *Fagus sylvatica* | 17 | 30 | 80 | - | 5.5 to 10.5 | 50 |
| *Fraxinus excelsior* | 14 | 30 | 50 | - | 4 to 9 | 50 |
| *Picea abies* | 12 | 28 | 200 | 2 | - | 50 |
| *Pinus sylvestris* | 12 | 28 | 130 | 4 | - | 50 |
| *Pseudotsuga menziesii* | 15 | 35 | 120 | 4 | - | 60 |
| *Quercus sp.* | 17 | 30 | 65 | - | 4.5 to 9 | 70 |
| *Sorbus aucuparia* | 14 | 30 | 50 | - | 4 to 9 | 50 |
| *Tilia sp.* | 14 | 30 | 50 | - | 4 to 9 | 50 |

The function by Liocourt ^5^ is described as :

$n_{i}=n_{1}q^{-(i-1)}$ (1)

Where: $n_{i}$: frequency of the ith diameter class; $n_{1}$: frequency of the first diameter class; $q$: coefficient of curve shape.

The function by Meyer ^6^ is as follows:

$n_{i}=ke^{ad_{i}}$ (2)

Where: $n_{i}$: frequency of the ith diameter class; $d_{i}$: diameter i; $a$: curve location parameter ; $k$: curve shape parameter.

S3-Table 2. Management regimes applying the crop-tree harvesting, for each development phase of the stand (young, growing and utilization phases). BAU stands for the Business-as-Usual thinning described in Table 1, in terms of the tending interventions, crop-tree number and target DBH. The crop-tree released was tailored for each species and species type, removing a fixed amount of competitors for conifers and applying a cleaning radius for broadleaves.

| Management | Young Phase | Growing phase | | | Utilization Phase |
| --- | --- | --- | --- | --- | --- |
|  | Tending | Crop-tree number | Conifer crop-tree release (N/crop-tree) | Broadleaf crop-tree release (radius m/10yr) | Target DBH (cm) |
| 1 | BAU | 0.75*BAU | 1 | 1 | BAU + 10 |
| 2 | BAU | 0.75*BAU | 1 | 1 | BAU |
| 3 | BAU | 0.75*BAU | 1 | 1 | BAU - 10 |
| 4 | BAU | 0.75*BAU | 2 | 1.5 | BAU + 10 |
| 5 | BAU | 0.75*BAU | 2 | 1.5 | BAU |
| 6 | BAU | 0.75*BAU | 2 | 1.5 | BAU - 10 |
| 7 | BAU | 0.75*BAU | 3 | 2 | BAU + 10 |
| 8 | BAU | 0.75*BAU | 3 | 2 | BAU |
| 9 | BAU | 0.75*BAU | 3 | 2 | BAU - 10 |
| 10 | BAU | BAU | 1 | 1 | BAU + 10 |
| 11 | BAU | BAU | 1 | 1 | BAU |
| 12 | BAU | BAU | 1 | 1 | BAU - 10 |
| 13 | BAU | BAU | 2 | 1.5 | BAU + 10 |
| 14 | BAU | BAU | 2 | 1.5 | BAU |
| 15 | BAU | BAU | 2 | 1.5 | BAU - 10 |
| 16 | BAU | BAU | 3 | 2 | BAU + 10 |
| 17 | BAU | BAU | 3 | 2 | BAU |
| 18 | BAU | BAU | 3 | 2 | BAU - 10 |
| 19 | BAU | 1.25*BAU | 1 | 1 | BAU + 10 |
| 20 | BAU | 1.25*BAU | 1 | 1 | BAU |
| 21 | BAU | 1.25*BAU | 1 | 1 | BAU - 10 |
| 22 | BAU | 1.25*BAU | 2 | 1.5 | BAU + 10 |
| 23 | BAU | 1.25*BAU | 2 | 1.5 | BAU |
| 24 | BAU | 1.25*BAU | 2 | 1.5 | BAU - 10 |
| 25 | BAU | 1.25*BAU | 3 | 2 | BAU + 10 |
| 26 | BAU | 1.25*BAU | 3 | 2 | BAU |
| 27 | BAU | 1.25*BAU | 3 | 2 | BAU - 10 |

S3-Table 3. Management regimes applying thinning from below. The percentages refer to the volume removal in relation to the standing stock in the three development phases of the stand.

|  | Young phase | Growing phase | Utilization phase |
| --- | --- | --- | --- |
| Management | Thinning (% of standing volume) | Thinning (% of standing volume) | Thinning (% of standing volume) |
| 28 | 30 | 10 | 50 |
| 29 | 30 | 10 | 60 |
| 30 | 30 | 20 | 50 |
| 31 | 30 | 20 | 60 |
| 32 | 30 | 30 | 50 |
| 33 | 30 | 30 | 60 |
| 34 | 40 | 10 | 50 |
| 35 | 40 | 10 | 60 |
| 36 | 40 | 20 | 50 |
| 37 | 40 | 20 | 60 |
| 38 | 40 | 30 | 50 |
| 39 | 40 | 30 | 60 |

S3-Table 4. Management regimes applying systems. For management regimes applying the Liocourt the target DBH was pscified and for management regimes applying the Meyer curve, the parameters k and a of Eq. 2 were speciefied.

|  | Liocourt | Management | Meyer | |
| --- | --- | --- | --- | --- |
| Management | Target DBH (cm) |  | Parameter k | Parameter a |
| 40 | 30 | 45 | 41.4 | 0.055 |
| 41 | 40 | 46 | 56.5 | 0.06 |
| 42 | 50 | 47 | 71.7 | 0.065 |
| 43 | 60 | 48 | 86.9 | 0.07 |
| 44 | 70 | 49 | 102.1 | 0.075 |

2 Data sources

For the initialization of the forest simulator Sibyla, the average dimeter, height and stock of each tree species are required as minimum inputs. With this information, the model is capable of automatically generate trees and tree coordinates based on the STRUGEN stand structure generator ^7^. We considered 0.16 ha simulation plots that were initialized using the forest inventory data base conducted by the Forest Administration in the state of Baden-Württemberg. This forest inventory, at forest office level, consists in a grid of plots with centers established every 200 x 200 m. The plots display a concentric design, with radius varying from 2 to 12 m, in which trees with DBH< 7 cm are recorded within a 2 m radius, 7 ≤ DBH < 15 cm are recorded within a 3 m radius, 15 ≤ DBH < 30 are recorded within a 6 m radius and DBH ≥ 30 cm are recorded within a 12 m radius ^8^.

For evaluating the stocking of each tree species in our area we assessed the forest development type of each stand and each plot recorded in the forest inventory. The forest development type refers to stands with similar initial status and objectives, which will be managed similarly and taking into account the multiple use of forests ^9^. We assessed the average condition, in terms of height, DBH and volume of each species in each forest development type, based on 2057 plots in our study region and ignoring tree species with low representability (volume inferior to 10 m³/ha). Subsequently, we assigned to the each stand in our research area, according to its forest development type, the corresponding species composition and stocking obtained from the forest inventory data for the model initialization.

References

1. Fabrika, M. Forest biodynamic simulator SIBYLA, conception, construction and program solution. Zvolen: Technická univerzita Zvolen (2005).
2. Fabrika, M., & Pretzsch, H. Forest Ecosystem Analysis and Modelling. Technical University in Zvolen: Zvolen, Slovakia (2013).
3. Hlásny, T. et al. Climate change impacts on growth and carbon balance of forests in Central Europe. Climate Research **47**, 219-236 (2011).
4. Pretzsch, H. Zum Einfluß des Baumverteilungsmusters auf den Bestandeszuwachs. Allg. Forst Jagdztg **166**, 190-201 (1995).
5. Liocourt F. De l'amenagement des sapinières. Bulletin Trimestriel, Société Forestière de Franche-Comté et Belfort, Julliet, 396-409 (1898).
6. Meyer, H. A. Structure, growth, and drain in balanced uneven-aged forests. Journal of forestry **50**, 85-92 (1952).
7. Pretzsch, H. Analysis and modeling of spatial stand structures. Methodological considerations based on mixed beech-larch stands in Lower Saxony. Forest ecology and Management **97**, 237-253 (1997).
8. ForstBW (Landesbetrieb Forst Baden-Württemberg). Aufnahmeanweisung permanente Betriebsinventur 2017 (2017).
9. ForstBW (Landesbetrieb Forst Baden-Württemberg). Richtlinie Landesweiter Waldentwicklungstypen. Stuttgart: Ministerium Ländlicher Raum Baden-Württemberg (2014).

Title page

Multiple uncertainties require a change of conservation practices for saproxylic beetles in managed temperate forests

Andrey L. D. Augustynczik*^1^, Rasoul Yousefpour^1^ & Marc Hanewinkel^1^

*Corresponding author ([andrey.lessa@ife.uni-freiburg.de](mailto:andrey.lessa@ife.uni-freiburg.de))

^1^Chair of Forestry Economics and Forest Planning, University of Freiburg, Tennenbacher Str. 4 (2. OG)

D-79106 Freiburg

Supplementary 4- Robust counterpart formulation

For the robust counterpart of our optimization problem, we applied an approach based on the framework proposed by Düzgün and Thiele ^1^. The authors developed a safe tractable approximation for binary optimization problems based on the safe tractable approximation framework developed by Ben-Tal et al. ^2^, namely the Bernstein approximation. The Bernstein approximation consists in establishing a bound on the perturbations based on moment generating functions, operating on the log-scale and using the exponential function as a generator. Given an uncertainty linear constraint of the form (Eq.1):

$z_{0}+\sum_{i=1}^{L} \zeta_{l}w_{l}\leq0$ (1)

$\ln(\text{E}e^{w^{T}\zeta})\leq\Phi(w)$ (2)

$\inf_{\beta>0} [z_{0}+\beta\Phi(\frac{w}{\beta})+\beta\ln(\frac{1}{\varepsilon})]\leq0$ (3)

where: $\zeta_{l}$: random perturbation; $\Phi(w)$: finite convex function with $\Phi(0)=0$; $z_{0}$: deterministic value; $\varepsilon$: error tolerance.

(Adapted from Ben-Tal et al. 2009).

We may establish a bound to the random perturbation with the help of generating functions (Eq. 2). The Bernstein approximation yields a solution to condition (Eq.3), that guarantees that the uncertain linear constraint (Eq.1) is respected with a probability of at least (1-$\varepsilon$). However, the condition (Eq.3) yields a non-linear relationship, which is computationally intractable for large MILP problems. In this sense, Düzgün and Thiele ^1^ recommend to choose an appropriate value for variable$\beta$ *a priori*, transforming the condition (Eq.3) into a linear one, and the robust optimization problem into a deterministic problem. The authors show that, given reasonable ranges for this variable, the optimal solution is fairly stable over a wide spectrum of $\beta$ values. Moreover, to overcome the limitation of assuming independent perturbations, the same authors suggest a modification of condition (Eq. 3) by applying the Cholesky decomposition of the covariance matrix of the uncertain data in place of vector$w$.

We based the robust counterpart of our optimization problem in the formulation proposed by Düzgün and Thiele ^1^ for a portfolio optimization problem. In this sense, our objective was the maximization of the Value-at-Risk (VaR) of the portfolio of management interventions at a confidence level of 5%. That means, in 95% of the realizations of the uncertain data, we expect that the total NPV obtained is higher than the VaR. To this end we changed the objective function of deterministic problem to one of the form (Eq. 4):

$\max Z=\text{E}NPV\text{-}F_{w}^{-1}(1-\varepsilon)$ (4)

Where: $\text{E}NPV$: expected NPV;$\text{F}_{w}^{-1}$: upper bound on the sum of NPV deviations.

For establishing the bound on the sum of NPV deviations, we considered a distribution of perturbations supported on [-1,1]. According to Ben-Tal et al. ^2^, the best function $\Phi(\cdot)$compatible with this information is the generating function of a two-point distribution:

$\Phi(w)=\ln(\sum_{l=1}^{L} \cosh(w_{l})+\mu\sinh(w_{l}))$ (5)

We used this function (Eq. 5) with a 0 mean, to compute the 95% quantile bound on the sum of NPV deviations. The deviations and expected NPV were computed based on the 400 deviates, derived from the 100 price, interest rate and forest disturbance realizations under each of the 4 climate change trajectories. For each management regime we computed the covariance matrix as follows:

$$\mathrm{cov}_{j}=\left[\begin{matrix} \text{E}(npv_{1}-\overline{npv}_{1})(npv_{1}-\overline{npv}_{1}) & ... & \text{E}(npv_{1}-\overline{npv}_{1})(npv_{582}-\overline{npv}_{582}) \\ ... & ... & ... \\ \text{E}(npv_{582}-\overline{npv}_{582})(npv_{1}-\overline{npv}_{1}) & ... & \text{E}(npv_{582}-\overline{npv}_{582})(npv_{582}-\overline{npv}_{582}) \end{matrix}\right] \forall j\in M$$

Where: $npv_{i}$: vector of NPV realizations.

We applied the Cholesky decomposition of the covariance matrix in order to establish a bound on the sum of deviations. Due to the fact that the deviations are activated only when the corresponding management is selected as part of the solution, the sum of deviations would generate a non-linear relationship when applied to function Eq.5. To overcome this issue we constructed a piecewise linear approximation of the function $\Phi$ in the interval [0,10]:

$L(\Phi(x))=\Phi(a_{0})+s_{0}(x-a_{0})+\sum_{k=1}^{m} \frac{s_{k}-s_{k-1}}{2}(abs_{k}+x-a_{k})$ (6)

(Adapted from Lin et al. ^3^)

$abs_{k}\geq x-a_{k} k=1...m-1$ (7)

$abs_{k}\geq-x+a_{k} k=1...m-1$ (8)

Where:$a_{k}$: breakpoint; $s_{k}$: slopes of line segments between breakpoints; $abs_{k}$: linearization of the term $\left|x-a_{k}\right|$.

Taking advantage of the convexity of the function$\Phi$, the approximation (Eq. 6), proposed by Li and Yu ^4^, allows a piecewise linearization without the need of extra binary variables, demanding however the addition of an auxiliary variable and a pair of constraints to each segment of the function to linearize the term $\left|x-a_{k}\right|$ (Eq. 7 and Eq. 8). For the linearization we used 16 breakpoints in the interval [0, 10], with a 0.1 step between in [0, 1], a 0.5 step in [1, 3] and a 7 step in [3, 10]. This construction yielded the best balance between accuracy and number of segments to be evaluated, as decreasing the number of breakpoints substantially decreased accuracy and the addition of extra breakpoints did not produce a significant increase in accuracy, at the cost of adding extra variables and constraints for each function evaluation.

For computing the final value of the parameter β in the robust objective function, we solved the robust optimization problem multiple times with different values for β, varying from 0.5 to 2.5 Million, with the best results achieved applying a β of 1 Million (Fig. 1).


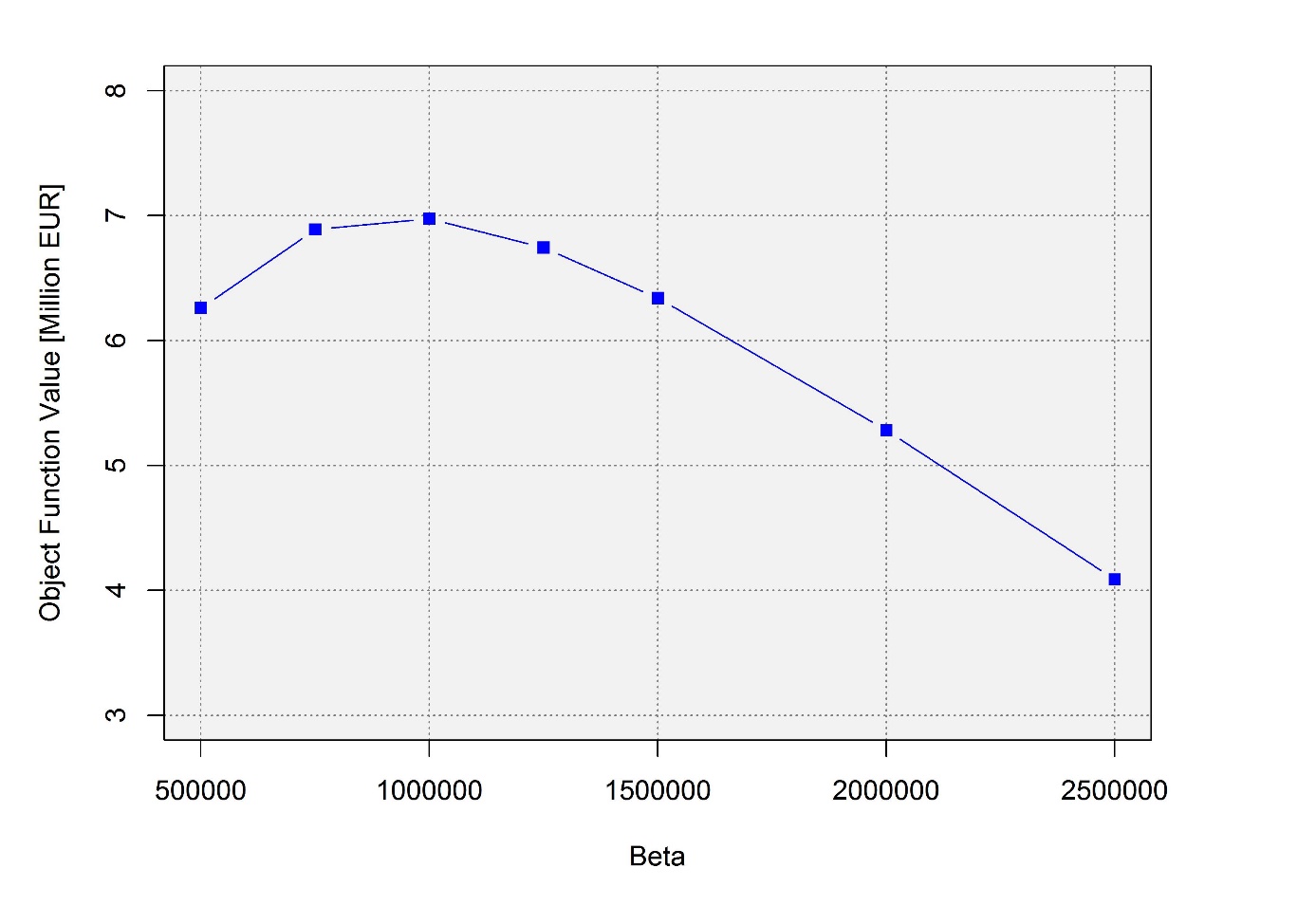


S4-Figure 1. The figure displays the objective function value with varying beta values for the robust optimization with all sources of uncertainty. The figure was created using RStudio 1.1.456 (https://www.rstudio.com/).

References

1. Düzgün, R., & Thiele, A. Robust binary optimization using a safe tractable approximation. Operations Research Letters **43**, 445-449 (2015).
2. Ben-Tal, A., El Ghaoui, L., & Nemirovski, A. Robust optimization. Princeton University Press (2009).
3. Lin, M. H., Carlsson, J. G., Ge, D., Shi, J., & Tsai, J. F. A review of piecewise linearization methods. Mathematical problems in Engineering, 2013 (2013).
4. Li, H. L., & Yu, C. S. A global optimization method for nonconvex separable programming problems. European journal of operational research **117**, 275-292 (1999).
